# Adaptive leadership in clinical encounters with women living with HIV

**DOI:** 10.1186/s12905-022-01810-1

**Published:** 2022-06-09

**Authors:** Schenita D. Randolph, Ragan Johnson, Kara McGee, Adaora A. Adimora, Catalina Ramirez, Donald E. Bailey, Lauren Holt, Amie Koch, Jacquelyn M. McMillian-Bohler, Tiarney Ritchwood, Michael V. Relf

**Affiliations:** 1 Present Address: Duke University School of Nursing, Durham, NC USA; 2 Sarah Graham Kenan Distinguished Professor of Medicine, Department of Epidemiology, School of Medicine, Gillings School of Global Public Health at the University of North Carolina at Chapel Hill, Chapel Hill, NC USA; 3 School of Medicine and the Project Director for the Women’s Interagency HIV Study at the University of North Carolina at Chapel Hill, Chapel Hill, NC USA; 4 Department of Family Medicine and Community Health, Duke University School of Medicine, Durham, NC USA; 5 Duke Global Health Institute, Durham, NC USA

**Keywords:** Women living with HIV (WLHV), Adaptive leadership framework, Clinical encounter

## Abstract

**Background:**

Women living with HIV (WLWH) report low engagement in health care, missed office visits, and less engagement in the clinical encounter. Strengthening the clinical encounter for WLWH may improve health outcomes and quality of life. The Adaptive Leadership Framework for Chronic Illness offers specific adaptive leadership strategies for providers to improve patient-provider interactions. The purpose of this study was to examine adaptive leadership behaviors that contribute to the development of effective patient-provider communication from the perspectives of WLWH.

**Methods:**

The descriptive, cross-sectional and qualitative study conducted interviews with 22 WLWH to assess perceptions of the clinical encounter related to HIV-related stigma, engagement in care, medical distrust, and experiences with discrimination and quality of life. Members of the study team using a set of a priori codes analyzed data using NVivo 12.0.

**Results:**

Participants described two primary themes and subthemes of each for adaptive leadership behaviors. The primary theme for adaptive leadership of providers was “my provider cares about me”; subthemes were communication, trust building takes time, and supportive providers are trusted. The primary theme for adaptive leadership of WLWH themselves was “I care about me; subthemes were self-advocacy and self-empowerment.

**Conclusions:**

Providers can use adaptive leadership behaviors during clinical encounters to support WLWH, improve patient-provider communication, enhance trust, and improve patient outcomes.

## Introduction

In 2018, there were 37, 968 new HIV diagnoses in the United States with 19% (7,190) being among women of which the majority (57%, 4097) were among Black women [1]. Patient perceptions of the quality of health care delivery during clinical encounters contributes to racial and ethnic disparities in health, including in HIV treatment and adherence for women living with HIV (WLWH) [2, 3].

The clinical encounter is the point at which transactions between patients and professionals take place and provides an opportunity for providers and health systems to influence the health of women vulnerable to and living with HIV [4–7]. When they identify with groups that have been historically disempowered, such as women from diverse racial and ethnic backgrounds and women living in poverty, WLWH are more likely to report lower engagement in HIV-related and other health care, miss more office visits, and be less engaged in the clinical encounter [2, 8, 9]. Such disparities contribute to low treatment adherence, lack of viral suppression, and overall outcome disparities [10, 11]. Among the complex factors that contribute to these disparities, patient-provider interactions during the clinical encounter have a strong impact on patient perception of health care quality, which ultimately influences health outcomes among WLWH [2, 12, 13].

Factors that influence the clinical encounter include the strength of patient-provider relationship, communication, and the degree of medical distrust. Poor patient-provider relationships and communication may be linked to poor health outcomes, particularly for Black women and other communities of color [12, 14]. Medical distrust – “a belief that we should question one’s motives and view their actions with suspicion because they are likely to act in a way that the quality of care or the accuracy of information provided may be compromised” [15, p. 442]—is negatively correlated with perceived need for medication and medication adherence, especially for Black people living with HIV [10]. Medical distrust is also linked to internalized stigma among WLWH and decreases their engagement in care [11, 16].

Nevertheless, some studies have highlighted women’s resiliency and advocacy for their own health, even while they are operating in systems where poor patient-provider interactions exist [17, 18]. In one study, Black female patients who reported perceived racial bias and lack of effective provider communication in the clinical encounter were motivated to be proactive self-advocates in the clinical setting [17]. Thus, both patients and providers contribute to effective clinical encounters and optimal health outcomes and quality of life.

### Adaptive leadership framework

Chronic illness presents challenges to patients, their families, and their providers that require adaptive leadership to support the clinical encounter. The Adaptive Leadership Framework for Chronic Illness proposes two broad categories of challenges that patients face – technical and adaptive [19, 20]. Technical challenges have clear solutions delivered by providers, such as prescribing an appropriate medication regimen. Adaptive challenges require the patient to engage in the work of acquiring new knowledge, learning a new skill, and/or changing their thinking and behavior, thus adapting to their new diagnosis [21]. Providers exhibiting adaptive leadership both galvanize patients to action and support patients and their families to do their adaptive work [21].

Previous research on the Adaptive Leadership Framework for Chronic Illness has largely focused on the provider’s perspective of the patient-provider relationship [4, 22]. However, a patient’s adaptive leadership skills are equally critical in a collaborative model of care in which the patient, family, and provider are actively engaged together in care [21]. Collaborative work requires the provider and patient to co-develop appropriate and sustainable approaches to care [20]. The provider’s role is to recognize the need for adaptive work, coupled with the ability to encourage, facilitate, or create adaptive change [20].

The Adaptive Leadership Framework for Chronic Illness can be helpful in identifying the perspectives of WLWH regarding specific behaviors that support effective collaborative patient-provider interaction. The purpose of this paper is to describe perspectives of WLWH regarding which adaptive leadership behaviors contribute to the development of successful patient-provider communication and clinical encounters.

## Methods

### Design and sample

The design of this study was descriptive, cross-sectional and qualitative. Participants were recruited from the North Carolina cohort of the Women’s Interagency HIV Study (WIHS), (a multi-center, prospective, observational cohort study of 123 eligible women living in the United States who have contracted HIV or vulnerable to HIV acquisition), which was designed to investigate the progression of HIV disease in women [23].

Eligibility criteria for the current study included age 18 or older, spoken English, and the ability to provide written informed consent. A study coordinator of the WIHS study team introduced the study to all eligible participants. Interested participants were asked for their preferred contact format (phone call, email, SMS, or US mail), which was forwarded to the PI who then contacted each woman to explain the study and answer any questions. Women who agreed to participate were scheduled for an interview.

Interviews were conducted in a private room at one of two clinical locations by the PI (MVR) between July and October 2016 and recorded using two encrypted digital recorders. The encrypted digital recorders were placed in a locked box, transported back to the study office, and secured in a double locked file cabinet in the principal investigator’s private office at the end of each interview day. Within 24 h, each interview was downloaded from the encrypted digital recorders to a study folder on a self-encrypting drive (SED). Interviews were transcribed verbatim by a doctoral student in nursing and verified as accurate by the study’s PI; the original digital recording was destroyed. Although saturation was identified by the seventeenth interview, a total of 22 interviews were conducted to ensure informational redundancy and theoretical saturation. Women participating in the study received a $50 gift certificate for participation.

### Ethical considerations

The Duke University Institutional Review Board (IRB # Pro00067256) provided study approval. All methods were performed in accordance with the relevant guidelines and regulations. All study participants were read the consent form, given an opportunity to have any questions addressed, and advised that participation was voluntary. Written informed consent was obtained on all study participants. This study utilized unique identification numbers to help protect the confidentiality of study participants; participant names were not used on any study related forms except for the written informed consent form. Forms with identifying information were maintained separately from data files in a secured file cabinet located in the private office of the principal investigator. Only the project director and the principal investigator had access to the file that linked the unique ID number to the name of the study participant; this file was password protected and stored in a separate study file on a secured electronic drive.

### Measures

Three unfolding stories of WLWH were developed by the PI for this study to facilitate the interviews. The illustrative stories depicted WLWH at three different ages starting with a woman in her 60’s, age 50 and between the ages of 20–30 years. The questions embedded within these stories were designed to explore the challenges of being an older WLWH. The probes were designed to have WLWH reflect on various challenges including aging, engagement in health care, health problems, family and intimate relationships and quality of life among older women [16]. As the interview progressed women were asked to reflect on what they thought these types of challenges would be for a woman in younger and middle-aged stages of life. The stories were designed to help the participants reflect on their lives and anticipate their futures to generate additional perspectives that a single interview could not embody. Questions were embedded within the stories to prompt descriptions of the experience of being a WLWH. Questions were related to HIV-related stigma, disclosure, antiretroviral therapy adherence, engagement in care, medical distrust, experiences with discrimination, depressive symptoms and quality of life. The interview guide developed for this study has been previously published [24].

### Data analysis

Each verbatim transcript was coded, using NVivo 12.0, by two members of the study team using a set of a priori codes. The codebook was enhanced with secondary codes that included HIV stigma and disclosure, the HIV care continuum, including engagement in care and ART adherence; medical distrust; experiences with everyday discrimination; and depressive symptoms. The coding team members also discussed emerging codes during regular team meetings. New codes were added to the codebook in an iterative manner. The revised codebook was used to code all transcripts.

Upon completion of initial coding, the team convened to discuss emerging themes in the context of the Adaptive Leadership Framework for Chronic Illness, during which areas of developing thematic foci were identified. To ensure credibility and trustworthiness of findings, the team broke into theme-based work groups and re-immersed themselves in the data to explore the richness of a specific theme and to seek confirmability of the findings. Analyst triangulation, triangulation of sources, and use of a single interviewer with experience in research and practice among WLWH facilitated credibility of findings.

## Results

**Fig. 1:**
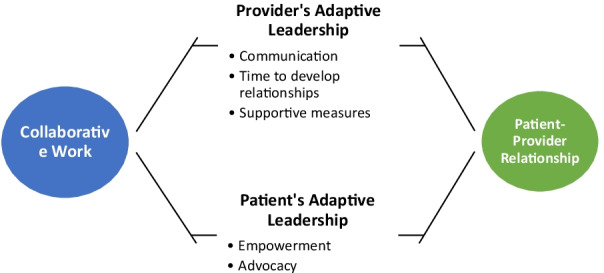
Collaborative adaptive leadership behaviors that enhance the patient-provider relationship

The mean age of participants (n = 22) was 52.2 years (range 38–62 years); 31.8% (n = 7) were younger than 50, and 86% (n = 19) self-identified as African American/Black. Participants described multiple health encounters with professionals within the health care system, including interactions with physicians, social workers, and nurses. We identified a primary theme and multiple subthemes of the participants’ perspectives on adaptive leadership behaviors of, each, their providers and themselves (Fig. 1). The primary theme for adaptive leadership of providers was “my provider cares about me”; subthemes were communication, trust building takes time, and supportive providers are trusted. The primary theme for adaptive leadership of WLWH themselves was “I care about me; subthemes were self-advocacy and self-empowerment.

### Adaptive leadership of providers

*My provider cares about me* Participants confirmed that a central adaptive challenge for patients is distrust in the healthcare system that negatively affects the relationship between themselves and their providers. WLWH described specific providers’ skills, attitudes, and behaviors that would exhibit adaptive leadership in gaining the trust of WLWH, therefore improving the patient-provider encounter. The primary theme that emerged from WLWH was that they trust providers who they feel care about them and their health. An example was sitting down and spending time with the patient. One participant stated: “for one thing, when she comes in the room, that it’s not a rush job, or you just a number, and I am trying to get in here and get out. I don’t care if she got 50 patients after you, she takes her time and sit and hear everything that is going on. (014)”.

The provider’s appropriate use of non-verbal communication indicated that the provider was trustworthy and that WLWH felt valued, heard, and cared for by the provider. One participant stated, “what I’m thinking is when there’s one on one, the eye contact. The body language of, I am paying attention to you, I hear you. Not just treating the patient like they are just a number but really focusing and listening to them. (010)”.

Women also discussed the importance of the provider getting to know them personally.

My doctor started calling me randomly, and we would just talk. Not just about the HIV, but about anything -- like a friend. It was helpful to me. She didn’t have to do that. (013)

Another described a personalized approach as,

You have to have a personal aspect to it. You can’t treat people like a textbook. And that’s how you know some professor you know they try to tell you know textbook like this like that. When am personal, I need that personal level. I need you to interact with me on a personal level. Show me some other side you know. Be professional but show me that personal. If it’s nothing more, when I bring my children, interacting with my children. (007)

*Communication* The WLWH felt cared for when providers were nonjudgmental and listened to their concerns. When asked what builds trust, one participant responded:

“Talk to them. If they have dealt with other patients, I’m sure they have heard different stories. This is nothing new, not a new thing to them. They should be able to relate to what that person is going through, let that person speak freely without judgment, and they should just listen. (001)” Another WLWH shared,

when I [was] sent to go talk to one of the therapists and one of the social workers that was at my doctor’s office, she made me feel so comfortable. And she made me feel like, she gave me her understanding of what I was dealing with at that time. She was clear, straight to the point, relatable. She didn’t look outside and judge me, she listened to me before she ever you know came to her own opinion. And then after listening to me and after being around me she was real confident in who I was as a person, she didn’t pre-judge me. (007)

WLWH appreciated the use of encouraging language, particular in self-care: “encouraging to that particular person to be involved in their own care.” Another woman shared that she felt comfortable communicating with her provider about her care, “, If I have a complaint about you, I’m a bring it up to you. This is what you’re doing that I don’t like. Could we figure out a way to do it differently? (014)”Such an approach creates space for shared decision- making and patient empowerment, which is essential to person-centered care.

*Trust building takes time* Participants often mentioned the importance of maintaining continuity with the same provider over time in order to build and maintain a trusting relationship. One woman stated, “Well, you definitely need a doctor that knows about you that you stick with, that you stay with that at any time you can go to, he knows your history. (004)” Another woman shared, “For those that don’t trust that it honestly takes time. It really does takes time, even with the forms and a trustworthy atmosphere, when they feel deep inside there’s no trust or they can’t trust, it just takes time, and it takes patience. (010)” Once developed, trust can be long lasting. One woman stated,

We need to truly feel like you have our well-being in mind. That’s the reason why I’ve had the same doctor for nine years. Because the first time I went to them, they developed a bond and trust with me that, they did the extra mile. Just to call me and tell me ok, how I am doing today? Let’s do this this way, am calling to check on you. (007)

Patients trust providers who consistently take the time to develop authentic relationships.

*Supportive providers are trusted* Women reported that their provider’s supportive response to their emotional distress regarding the HIV diagnosis was an important part of building a trusting relationship. One participant described her provider’s response,

She was like; it’s not a death sentence like it used to be. So, she reminded me of that. It’s not a death sentence. So, it’s not like gonna die, you probably gonna die of whatever, natural causes, somewhere down the road. It won’t be this. So she reminded me of that, and that was helpful. Even though I knew I wasn’t gonna die, even still just her saying that it was still helpful. So it made me more focus on, well, how am I going to live with this. (006)

Participants also discussed how providers display adaptive leadership by assuring that their patients have the support they need to cope with and manage living with HIV. When asked about how WLWH can get help, one participant discussed the role of having support from a counselor,

They need to know that somebody is there, and they are not by themselves. The longer a person who is on the shameful closet mood, the longer it is they will stay there and the longer it will be go get them out. So, I feel like in the beginning to have someone that you can connect to. If they are not comfortable with a peer, like I said, a counselor. I had a counselor, I had somebody that, if I didn’t want to talk to anybody, I could talk to her. In the beginning, you don’t want to talk to anybody but the person who shows forth a little bit of caring. A little bit of support is a small anchor towards pulling that person in. (010)

This participant also suggested providers should “connect a newly-diagnosed person with different resources that are connected to them. (010)” Another shared the importance of having a support group to address feeling overwhelmed by living with HIV,

If it’s nothing else but having support groups. Places where you are meeting other people with the virus. [name of support group] honey it does me a world of good. I am looking at everybody, and I am like wow I am seeing people that I see on a everyday basis that I didn’t know that was HIV positive. I know we all keep our confidentiality or whatever, but hey, and then not only that but the people who do the volunteer services…. But, it’s like saying everybody is ok. Get stuff like that going. We have parties, Christmas parties, stuff like that they do stuff like that. And when to be encouraging for them to show up. So once you get around other people, your breathing is not so heavy. I use that analogy a lot but just breathe, it’s ok. It’s ok. Helps me to remind me that I am not by myself. (018)

Providers’ familiarity with culturally relevant resources facilitates adaptation. One participant stated that recommended resources should be based on,

whether it be a younger woman or an older woman. Some people are very specific about ethnicity. They don’t connect to, so I am a mother and a grandmother. I’ve taken care of and I wasn’t that person who partied hard or did drugs. So maybe I don’t fit in that category. Maybe separate themselves but maybe what the person can do is connect them to different resources that would benefit them for what they are looking for. (010)

### Adaptive leadership of patients (WLWH)

*I care about me* Patients demonstrate adaptive leadership in the healthcare environment by caring about their own health and well-being. This behavior was inherent in some of the women and learned in others. One woman stated, “…it took me a long time to realize that, if I couldn’t stand up for myself and fight for myself, I wasn’t going to make it. (002)” But all of the women in the study described the challenge of facing their diagnosis. One woman shared, “So it’s a choice that I have to make, I can get up today and show up for life. (018)” More specifically, participants described the skills that contributed to self-care and care of their relationships to providers as self-advocacy and self-empowerment.

*Self-advocacy* The importance of speaking up if something was bothering her was a consistent theme, such as,

yeah you can’t be closed-mouth, because a closed-mouth don’t get you anywhere. If you tell them exactly what is on your mind and how you feel, you don’t have to use big words or sugar coat it, just tell them how you feel, what’s on your mind. If you don’t like something, “I don’t like this, I don’t wanna do this.” Let’s find something else, another way to do it, you just have to let them know. (012)

When asked about how a woman living with HIV could advocate for herself, one participant responded, “Don’t be afraid to ask questions, don’t be afraid to be bold with your provider because that is what they are there for. (016)” Another participant said, “You are allowed to change doctors anytime you wish. So you know if you are having problems with one change doctors. That’s what I would tell her in her instance, if you feel like your doctor not treating you fairly, ask for another one (014)”.

*Self-empowerment* Self-empowerment and self-compassion also helped women live with HIV, and an innate self-empowerment ensured safety in the patient-provider relationship. When participants experienced providers whom they could not trust, they were empowered to make another choice. One participant stated that after she felt her provider was giving her a

cold shoulder…. They don’t tell what you need to know. You do have a choice. When my next appointment came I said, ‘No I don’t want her. Give me another one. (001)

Another woman agreed, stating,

It basically comes down to whether I think you have my best interest at heart. And the minute that I think don’t have my best interests at heart, I am out the door. And I have had that happen you me on a couple of occasions when I can think of. (006)

With respect to the adaptive challenge of discrimination and/or racism from the provider, women felt empowered to advocate for themselves. When asked what skills a person needs to feel empowered when confronted with discrimination, one woman shared,

Trying to address it directly with that particular physician, that can be a step that would be the most important step, to let them know because maybe they don’t even realize that they are treating the person that way. And then the next from there if step one doesn’t work with speaking with the physician, maybe go to a higher level. At that point, especially if it is like a very small town and it’s rural and it’s really all they have, it’s not much that they can do. (010)

Providers and health care facilities have a responsibility to inform patients of their rights, thereby improving their confidence. The previous participant went on to state that every patient should be

…informed at the very beginning of their rights and responsibilities. Give them documentation, not everybody keeps paperwork but ok at least that was presented to them so they know that. And I would say periodically still inform them of that even if it is every visit to remind them that you have rights so that they have rights and responsibilities and stuff like that. (010).

WLWH participants believed that an important step to empowerment was to educate themselves and ask for information about HIV. One woman shared, “They are there to help me, and that’s what I look at them as. Give me the right information so I can take care of myself (009).”While the provider is a resource, WLWH also expressed that educating themselves increased confidence in their own health. One participant stated,

Just wanting to be a healthier person, you know being educated, you know education is the key. You know you can’t see something and say, you know you have to be informed. Education you know. Be willing to learn, and you know you got to do it for yourself, ain’t nobody going to take care of you but you. It’s your responsibility to take care of your health, and until you get on board and decide that’s what you want to do, nobody can make you do. (004)

## Discussion

Findings from this study highlight the needs and capacities of women living with HIV to achieve meaningful, collaborative relationships with their HIV providers. Both the WLWH and the providers had technical skills and capacities to build upon in developing person-centered therapeutic relationships and warrant critical examination. The Adaptive Leadership Framework for Chronic Illness [20] offered a framework for exploring factors that improve clinical encounters from the patients’ perspective [21, 22].

This framework has been used in the context of other chronic conditions, including diabetes, [25] male infertility [26] and Hepatitis C [21]. We extended those studies, finding that partnership with the provider, trust, positive communication, and provider support are key elements of adaptive leadership during HIV care. Because WLWH are faced with challenges including intimate partner violence, sexual and reproductive health issues and have complex adaptive challenges, such as stigma, disclosure difficulties, and mental health comorbidities, providers have a responsibility to address these challenges in addition to the technical challenges of prescribing anti-retroviral medications and evaluating biomarkers of treatment effectiveness. Black, Indigenous, and People of Color living with HIV may have additional challenges related to medical distrust, social determinants of health, and intersectional stigmas including racism and sexism. This study showed that the provider as an adaptive leader can implement strategies to improve trust, such as providing continuity in care, communicating with WLWH outside of the clinical encounter, allotting time during visits to explore how the WLWH is doing in general, and collaborating with them in shared decision-making.

WLWH demonstrated a sense of self-empowerment and were active participants in their care. The historical and cultural social construct of the ‘Strong Black Woman’ includes self-efficacy and may cross-pollinate to health. Consistent with Brody et al., [27] WLWH in our sample were able to advocate for themselves, but none provided perspectives for effecting social and structural level changes to the healthcare system. Additional research is needed in this area, as such perspectives may inform the dismantling of racism in healthcare systems.

Consistent with Dale and Safren’s [28] research among Black WLWH, we found that WLWH described nurturing provider-client relationships as those that consisted of honesty, trust, non-judgmental views, positive regard for the women’s well-being, and genuine concern. Dale and Safren [28] found that positive trusting relationships with the provider translated to adaptive and technical work such as appointment attendance, medication adherence, and improved mental and physical health care. To the degree that WLWH have additional adaptive challenges of limited family, financial, or emotional support, providers can show adaptive leadership by connecting WLWH to culturally relevant support groups, peer support, and other tangible resources (legal, transportation, housing). These actions may further enhance WLWH’s resilience [28]. It is also noteworthy that several participants valued providing social support for others in their communities. The potential for creating and enhancing culturally relevant networks of support that focus on individual advocacy for others and peer advocacy in healthcare encounters is an area for future research.

### Limitations

This study has some limitations. Although the team was immersed in the analysis and coding to increase validation of findings, study participants did not provide feedback on the study’s results and did not review the study transcripts. Future studies should include WLWH throughout the research process from conceptualization to dissemination to ensure participants validation of findings as well as input for next steps. Furthermore, participants in this study were from the Southeastern United States, therefore generalizability to women in other geographic regions of the US and countries should be done with caution as their experiences may differ. Finally, the length of time the participants had been living with HIV was not collected. Future research should consider this variable and its impact on experiences in the clinical encounter over time.

## Conclusions

Raising awareness about the perspectives of WLWH on the roles providers play in the broader health care system, specifically in HIV prevention and care, is essential. An awareness of women’s perspectives is a first step toward modification of provider behaviors and institutional practices that can then lead to better health outcomes for women living with HIV. Awareness of and implementation of adaptive leadership skills is an important approach in assuring positive clinical encounters for WLWH and can lead to better health outcomes by improving retention, treatment and satisfaction with care.

## Data Availability

The datasets generated and/or analyzed during the current study are not publicly available due to the sensitive nature of this data and the potential risk of participant identification. Data are available from the corresponding author on reasonable request.

## References

[1] Centers for Disease Control and Prevention. HIV Surveillance Report 2018 (Updated); 2020. Accessed May 17, 2021. http://www.cdc.gov/hiv/library/reports/hiv-surveillance.html

[2] FitzGerald C, Hurst S (2017). Implicit bias in healthcare professionals: a systematic review. BMC Med Ethics.

[3] Rice WS, Fletcher FE, Akingbade B (2020). Quality of care for Black and Latina women living with HIV in the U.S.: a qualitative study. Int J Equity Health.

[4] Bailey DE, Muir AJ, Adams JA (2019). Clinical encounters and treatment initiation for chronic hepatitis C patients: applications of adaptive leadership framework for chronic illness. SAGE Open.

[5] Blair JM, Fagan JL, Frazier EL (2014). Behavioral and clinical characteristics of persons receiving medical care for HIV infection - Medical Monitoring Project, United States, 2009. MMWR Suppl.

[6] Castle B, Wendel M, Kerr J, Brooms D, Rollins A (2019). Public health’s approach to systemic racism: a systematic literature review. J Racial Ethn Health Disparities.

[7] Hall WJ, Chapman MV, Lee KM (2015). Implicit racial/ethnic bias among health care professionals and its influence on health care outcomes: a systematic review. Am J Public Health.

[8] Fiscella K, Boyd M, Brown J (2015). Activation of persons living with HIV for treatment, the great study. BMC Public Health..

[9] Blackstock OJ, Beach MC, Korthuis PT (2012). HIV providers’ perceptions of and attitudes toward female versus male patients. AIDS Patient Care STDs.

[10] Pellowski JA, Price DM, Allen AM, Eaton LA, Kalichman SC (2017). The differences between medical trust and mistrust and their respective influences on medication beliefs and ART adherence among African-Americans living with HIV. Psychol Health.

[11] Harris OO, Leblanc N, McGee K, Randolph S, Wharton MJ, Relf M (2020). Alarm at the gate-health and social inequalities are comorbid conditions of HIV and COVID-19. J Assoc Nurses AIDS Care JANAC.

[12] Beach MC, Saha S, Korthuis PT (2011). Patient–provider communication differs for black compared to white HIV-infected patients. AIDS Behav.

[13] Boehme AK, Moneyham L, McLeod J (2012). HIV-infected women’s relationships with their health care providers in the rural deep south: an exploratory study. Health Care Women Int.

[14] Zhang C, McMahon J, Leblanc N, Braksmajer A, Crean HF, Alcena-Stiner D (2020). Association of medical mistrust and poor communication with HIV-related health outcomes and psychosocial wellbeing among heterosexual men living with HIV. AIDS Patient Care STDs.

[15] Griffith DM, Bergner EM, Fair AS, Wilkins CH (2021). Using mistrust, distrust, and low trust precisely in medical care and medical research advances health equity. Am J Prev Med.

[16] Relf MV, Pan W, Edmonds A, Ramirez C, Amarasekara S, Adimora AA, Discrimination (2019). Medical distrust, stigma, depressive symptoms, antiretroviral medication adherence, engagement in care, and quality of life among women living with HIV in North Carolina: a mediated structural equation model. J Acquir Immune Defic Syndr 1999.

[17] Randolph SD, Golin C, Welgus H, Lightfoot AF, Harding CJ, Riggins LF (2020). How perceived structural racism and discrimination and medical mistrust in the health system influences participation in HIV health services for black women living in the United States South: a qualitative, descriptive study. J Assoc Nurs AIDS Care.

[18] Sacks TK (2018). Performing Black womanhood: a qualitative study of stereotypes and the healthcare encounter. Crit Public Health.

[19] Heifetz RA, Linsky M, Grashow A (2009). The Practice of Adaptive Leadership: Tools and Tactics for Changing Your Organization and the World.

[20] Anderson RA, Bailey DE, Wu B (2015). Adaptive leadership framework for chronic illness. Adv Nurs Sci.

[21] Bailey DE, Docherty SL, Adams JA (2012). Studying the clinical encounter with the Adaptive Leadership framework. J Healthc Leadersh.

[22] Thygeson M, Morrissey L, Ulstad V (2010). Adaptive leadership and the practice of medicine: a complexity-based approach to reframing the doctor–patient relationship. J Eval Clin Pract.

[23] Adimora AA, Ramirez C, Benning L (2018). Cohort profile: the women’s interagency HIV study (WIHS). Int J Epidemiol.

[24] Bailey DE, Caiola CE, Adimora AA, Ramirez C, Holt L, Johnson R, Koch A, McGee K, McMillian-Bohler JM, Randolph SD, Ritchwood TD, Relf MV (in press) Adaptive challenges, adaptive work, and adaptive leadership among women living with HIV in the Southern United States: findings from a qualitative study. J Assoc Nurses AIDS Care. 10.1097/JNC.0000000000000288 PMC9244859 35500057

[25] Carthron D, Bailey DE, Anderson R (2015). Adaptive challenges rising from the life context of African-American caregiving grandmothers with diabetes: a pilot study. Healthcare.

[26] Stevenson EL, McEleny KR, Moody E, Bailey DE (2019). Applying the adaptive leadership framework for chronic illness to understand how American and British men navigate the infertility process. Health Psychol Open.

[27] Brody LR, Jack DC, Bruck-Segal DL (2016). Life lessons from women with HIV: mutuality, self-awareness, and self-efficacy. AIDS Patient Care STDs.

[28] Dale SK, Safren SA (2018). Resilience takes a village: black women utilize support from their community to foster resilience against multiple adversities. AIDS Care.

